# Molecular characterization of eukaryotic algal communities in the tropical phyllosphere based on real-time sequencing of the 18S rDNA gene

**DOI:** 10.1186/s12870-018-1588-7

**Published:** 2018-12-18

**Authors:** Huan Zhu, Shuyin Li, Zhengyu Hu, Guoxiang Liu

**Affiliations:** 1 Key Laboratory of algal Biology, Institute of Hydrobiology, Chinese Academy of Sciences, Wuhan, 430072 People’s Republic of China; 2 State Key Laboratory of Freshwater Ecology and Biotechnology, Institute of Hydrobiology, Chinese Academy of Sciences, Wuhan, 430072 People’s Republic of China

**Keywords:** Aerial algae, Community structure, Diversity, Phyllosphere, Phylogeny, SMRT sequencing, Tropical forest

## Abstract

**Backgroud:**

Foliicolous algae are a common occurrence in tropical forests. They are referable to a few simple morphotypes (unicellular, sarcinoid-like or filamentous), which makes their morphology of limited usefulness for taxonomic studies and species diversity assessments. The relationship between algal community and their host phyllosphere was not clear. In order to obtain a more accurate assessment, we used single molecule real-time sequencing of the 18S rDNA gene to characterize the eukaryotic algal community in an area of South-western China.

**Result:**

We annotated 2922 OTUs belonging to five classes, Ulvophyceae, Trebouxiophyceae, Chlorophyceae, Dinophyceae and Eustigmatophyceae. Novel clades formed by large numbers sequences of green algae were detected in the order Trentepohliales (Ulvophyceae) and the *Watanabea* clade (Trebouxiophyceae), suggesting that these foliicolous communities may be substantially more diverse than so far appreciated and require further research. Species in Trentepohliales, *Watanabea* clade and *Apatococcus* clade were detected as the core members in the phyllosphere community studied. Communities from different host trees and sampling sites were not significantly different in terms of OTUs composition. However, the communities of *Musa* and *Ravenala* differed from other host plants significantly at the genus level, since they were dominated by Trebouxiophycean epiphytes.

**Conclusion:**

The cryptic diversity of eukaryotic algae especially Chlorophytes in tropical phyllosphere is very high. The community structure at species-level has no significant relationship either with host phyllosphere or locations. The core algal community in tropical phyllopshere is consisted of members from Trentepohliales, *Watanabea* clade and *Apatococcus* clade. Our study provided a large amount of novel 18S rDNA sequences that will be useful to unravel the cryptic diversity of phyllosphere eukaryotic algae and for comparisons with similar future studies on this type of communities.

**Electronic supplementary material:**

The online version of this article (10.1186/s12870-018-1588-7) contains supplementary material, which is available to authorized users.

## Background

The phyllosphere comprises the aerial parts of plants and is mainly constituted by the leaves, which can be considered an ephemeral environment [1]. Organisms inhabiting the phyllosphere often suffer an oligotrophic stress due to direct exposure to atmosphere. In tropical rainforests, the prevailing epiphytes living as inhabitants of the phyllosphere consist of various bacteria, lichens, fungi, free-living algae and bryophytes [2–4]. The microbiomes formed by these organisms often produce patches of different colors (i.e. green, grey-green, yellow or orange), mainly due to primary or secondary pigments of different algae or lichens.

Most studies on microbial life in the phyllosphere have focused on bacteria and fungi. The composition of their communities, their adaptions to the phyllosphere and multiple interactions have been investigated [1, 5–8]. However, the algal communities of the phyllosphere have been less intensively studied. It has been shown that these epiphyllous forms represent a very heterogeneous and evolutionarily diverse assemblage, which mainly consist of cyanobacteria and green microalgae such as coccoid trebouxiophycean algae and branched trentepohliacean algae [9, 10]. Since most algae are aquatic, these epiphyllous forms have generally received little attention in the past and their taxonomic and ecological knowledge has rapidly increased in recent years [11–15]. Cultivation-independent studies reported that eukaryotic algae are abundant on rainforest leaves, and the available knowledge about these forms suggests that there may be a huge number of unknown species [16–19]. Furthermore, it has been proved that coccoid green algae with identical or almost identical morphologies may be substantially different from the genetic point of view, and that single-celled coccoid microchlorophytes represent a polyphyletic complex of cryptic species [20–22]. Most coccoid green algae or SSU rDNA sequences retrieved from leaves are nested into trebouxiophycean clades corresponding to the genera *Chlorella*, *Auxenchlorella*, *Myrmecia*, *Coccomyxa*, *Elliptochloris*, *Diplosphaera*, *Stichococcus*, *Prasiola*, *Trebouxia* and *Asterochloris* [15, 23–28], whereas filamentous green algae belong to the genera *Phycopeltis*, *Cephaleuros*, *Trentepohlia* and *Klebsormidium* [9, 17, 29, 30]. Additionally, the Xanthophyceaen genus *Heterococcus* was also reported as phycobiont in lichens [26].

Given that phyllosphere microbiota play an unequivocal role in the functioning of forest ecosystems, it is remarkable how little we know about their diversity and distribution patterns [31]. It is therefore urgent to get a better characterization of the diversity of foliicolous algal communities. Some studies have investigated the taxonomy or diversity of several subaerial algal communities using morphological methods, and cultivation or various cultivation-independent molecular methods (i.e. clone library and Sanger sequencing) [14, 32]. The traditional approaches (e.g. morphological observation, and cultivation-independent molecular clone) are affected by several problems (i.e. low efficiency to get massive sequences, and some species difficult to grow in culture) and are inadequate to unravel the full diversity of these communities. The widely used high-throughput platforms (i.e. 454 and MiSeq) generate moderate to high numbers of high-quality short sequences, which are often not sufficient to obtain well-resolved results. The communities characterized by these platforms often suffer from low resolution of taxonomic coverage. The SSU rDNA gene is the molecular marker for which the largest amount of sequence data is available and is usually adequate for inference at genus level. Using nearly whole length SSU can provide a highly-resolved assessment of the taxonomic composition and diversity of communities.

In present study, we collected samples of 40 epiphyllous communities from the Xishuangbanna Tropical Botanical Garden (Yunnan, Southern China) in order to investigate comprehensively their diversity and characterize it with high resolution. We selected two pairs of primers to amplify the SSU sequences. In order to obtain well-resolved results, we used single molecular real-time (SMRT) to sequence our PCR products. Based on the results obtained, we assessed the taxonomic composition and molecular diversity of the 40 epiphyllous algal communities sampled.

## Results

### Taxonomic diversity across different phyllospheres

**Fig. 1:**
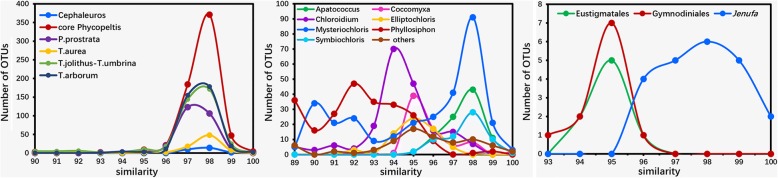
Similarity of most eukaryotic algae OTUs based on BLASTn analysis

After deletion of the ambiguous and low-quality sequences (unambiguous sequences less than 1500 bp long and chimeric sequences), we obtained a total of 152,324 high quality sequences. The frequency of the sequences lengths is plotted for the 152,324 high quality sequences in Additional file 1: Figure S1; most sequences were distributed at 1650 to 1850 bp length. We obtained 19,831 OTUs under 98% similarity cutoff level. The sampling intensity was evaluated. Random sub sampling was conducted for sequencing depths from 0 to 700 with steps of 100 sequences per sample. The rarefaction curve showed that our sequencing depth was sufficient (Additional file 2: Figure S2). A BLASTn was performed for annotation all 19,831 OTUs. Among them, 2922 were annotated as eukaryotic algae and mainly consisted of Trebouxiophyceae and Trentepohliales, with a very small part as Chlorophyceae (Volvocales and *Jenufa* clade), Eustigmatales and Gymnodiniales (Fig. 1). The present study was focused on eukaryotic algae, so we did not consider other microbial groups.

Sequences with the highest similarity to each OTU in GenBank were retrieved by BLASTn searches and then downloaded for further phylogenetic analysis. Finally, matrices with 1283, 1922, 194, 93 and 64 sequences respectively consisting of Trebouxiophyceaen, Trentepohliacean, Chlorophycean, Dinophycean and Eustigmatophyceaen OTUS, were used for phylogenetic analyses, in which Oedogoniales and Volvocales, Cladophorales, Oedogoniales, Perkinsus and Xanthophyceae were respectively used to select roots.

**Fig. 2:**
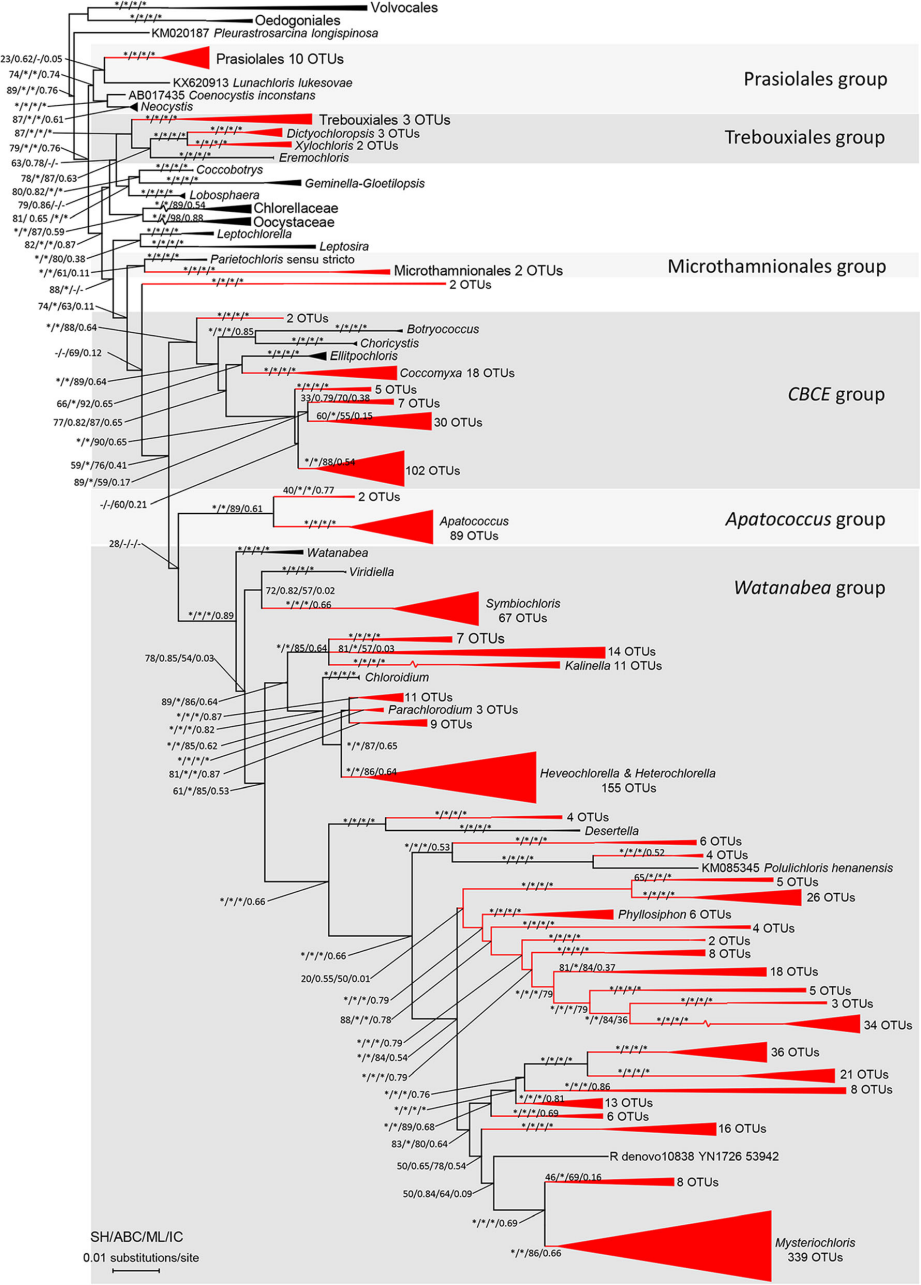
Maximum likelihood tree of Trebouxiophyceae showing phylogenetic relationships of Trebouxiophycean OTUs and sequences retrieved from GenBank (https://www.ncbi.nlm.nih.gov/genbank/). Branches with asterisks indicate statistical support from SH-test, aBayesian test, maximum-likelihood bootstrap and internodes certainty ≥90, 0.90, 90 and 0.90 respectively. The tree was rooted by Chlorophycean Volvocales and Oedogoniales, and 6 order-level phylogroup were shaded in grey. Clades with present OTUs were colored in red

Phylogenetic analysis showed that the Trebouxiphycean OTUs mainly belonged to 6 independent clades, i.e., Prasiolales clade, Microthamniales clade, *Apatococcus* clade, *Watanabea* clade (including *Mysteriochloris*, *Heveochlorella*, *Heterochlorella*, *Phyllosiphon*, *Polulichloris*, *Desertella*, *Kalinella*, *Parachloroidium* and *Symbiochloris* groups), Trebouxiales clade (including *Xylochloris*, *Eremochloris*, *Dictyochloropsis* and Trebouxiales) and CBCE clade (*Coccomyxa*, *Botryococcus*, *Choricystis* and *Elliptochloris*) clade. Our phylogenetic analysis of Trebouxiophyceae showed that the Trebouxiophycean OTUs belonged primarily to 43 clades (Fig. 2, red clades), of which 39 clades are well supported.

**Fig. 3:**
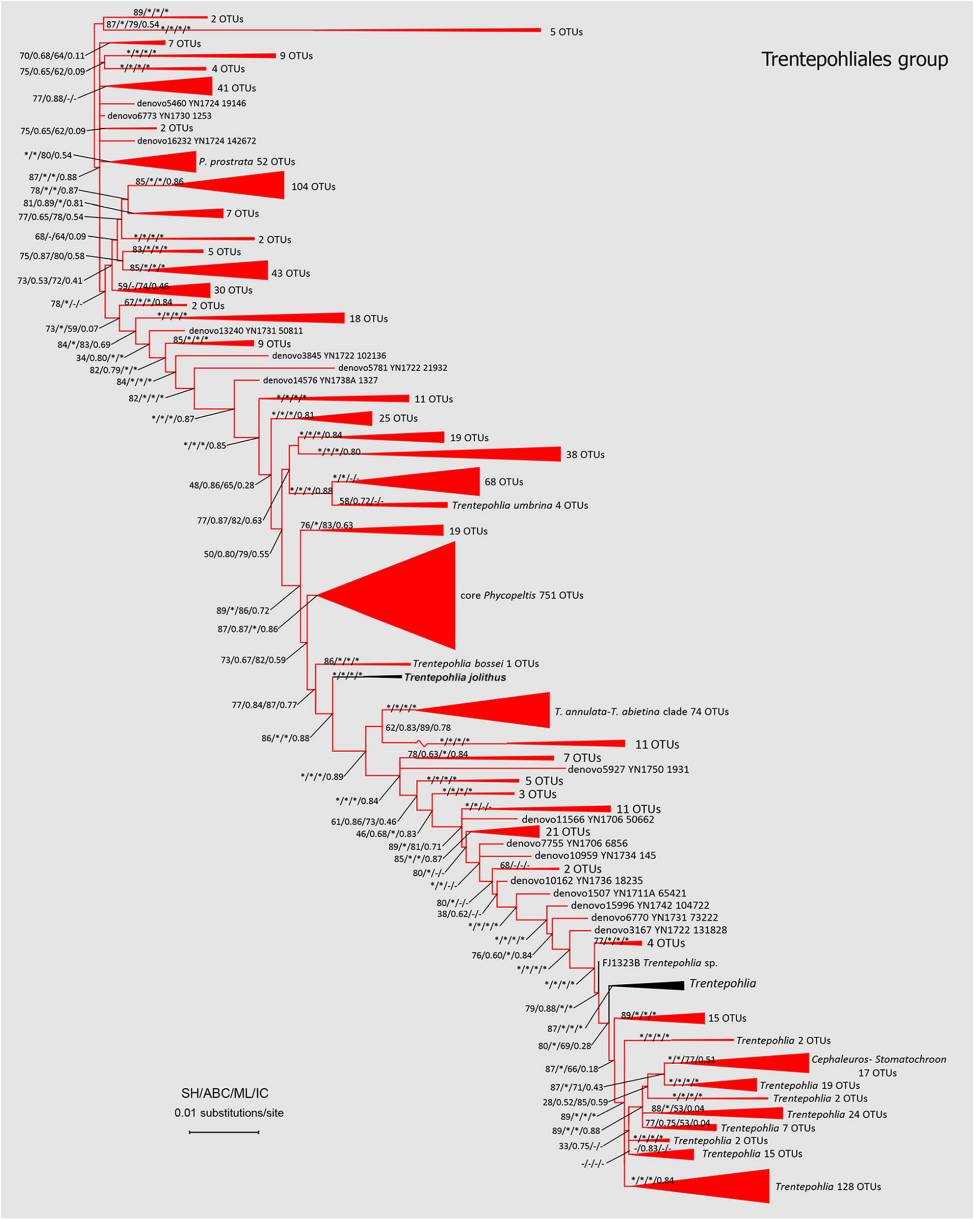
Maximum likelihood tree of Trentepohliales showing Phylogenetic relationships of new obtained Trentepohliacean OTUs and sequences retrieved from GenBank (https://www.ncbi.nlm.nih.gov/genbank/). Branches with asterisks indicate statistical support from SH-test, aBayesian test, maximum-likelihood bootstrap and internodes certainty ≥90, 0.90,90 and 0.90 respectively. The tree was rooted by the deep lineage recovered in phylogenetic analysis which Cladophorales were used as outgroup. Clades with present OTUs were colored in red

The topology obtained from Maximum-likelihood analysis of Trentepohliales is shown in Fig. 3. Besides major lineages (*Cephaleuros*, *Stomatochroon*, *Trentepohlia arborum* clade, *T. aurea*, *T. jolithus* clade, *T. annulata* clade, core *Phycopeltis*, *P. prostrata* clade et al.) recovered from previous studies [33, 34], we also designated several novel lineages. In present phylogenetic analysis, the genera *Trentepohlia* and *Phycopeltis* were still paraphyletic. With the focus on *Phycopeltis* and its relative clades, the core *Phycopeltis* clade may be still monophyletic with the richest OTUs, which is in accordance with our expectation. The unexpected result is several novel deep lineages recovered including previously defined *P. prostrata* clade. OTUs fall into those novel lineages have an identity with their closest relatives about 91 to 94%.

**Fig. 4:**
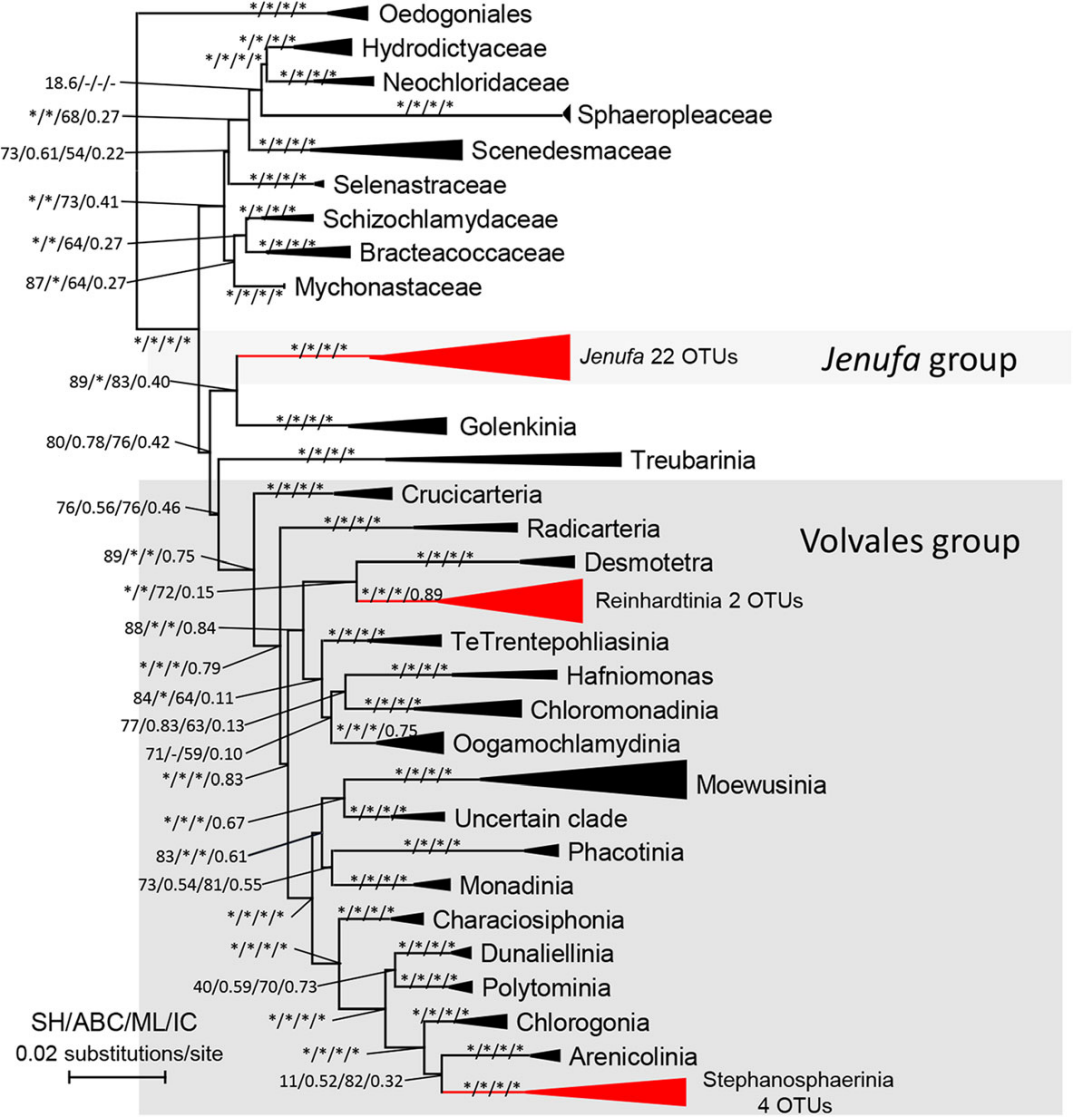
Maximum likelihood tree of Volvocales showing phylogenetic relationships of new obtained Volvocales and *Jenufa* clade OTUs and sequences retrieved from GenBank (https://www.ncbi.nlm.nih.gov/genbank/). Branches with asterisks indicate statistical support from SH-test, aBayesian test, maximum-likelihood bootstrap and internodes certainty ≥90, 0.90,90 and 0.90 respectively. The tree was rooted by Oedogoniales. Clades with present OTUs were colored in red

**Fig. 5:**
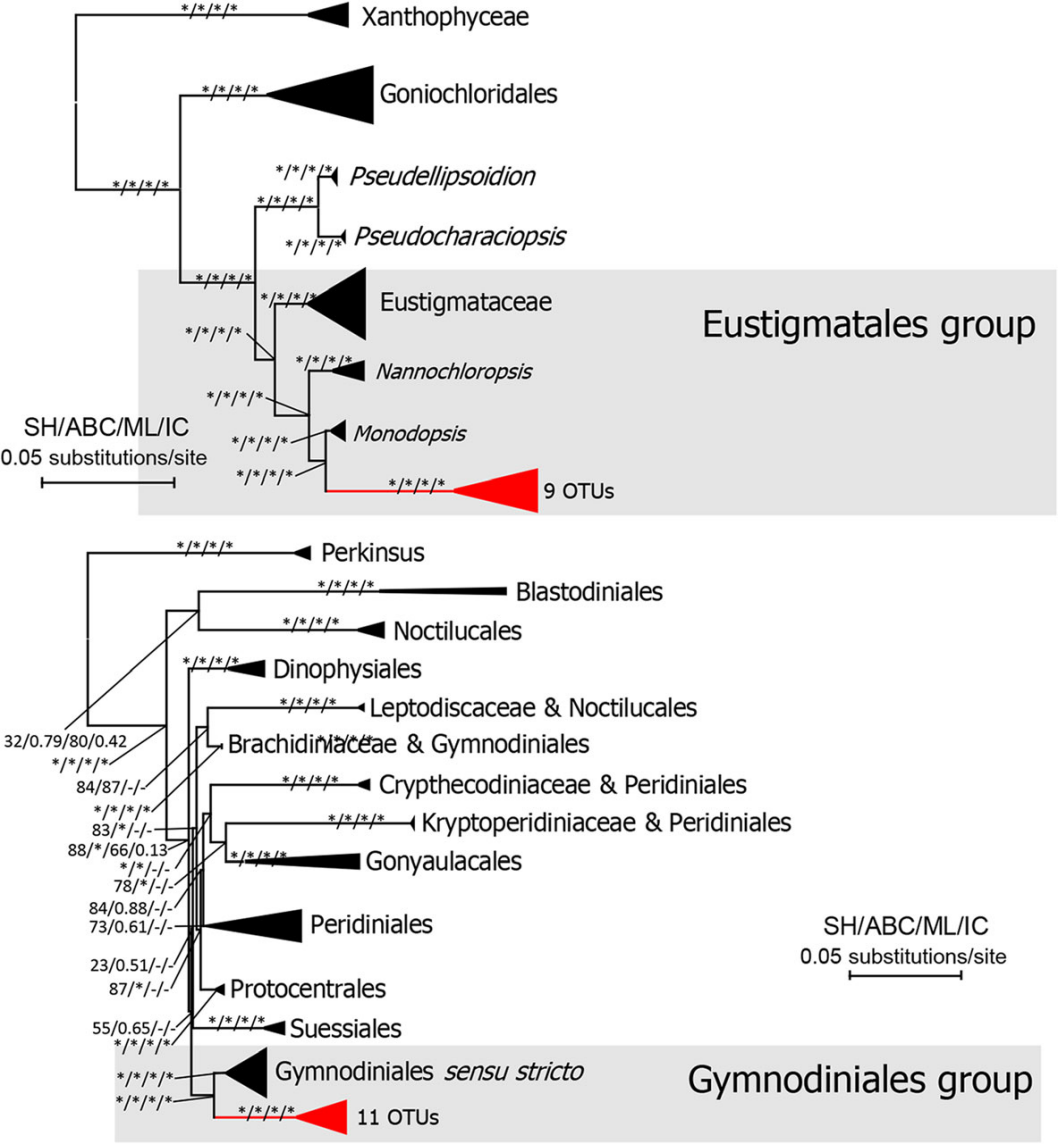
Maximum likelihood trees of Eustigomatophycea (A) and Dinophyceae (B) showing phylogenetic relationships of new obtained Eustigmatales and Gymnodiniales OTUs and sequences retrieved from GenBank (https://www.ncbi.nlm.nih.gov/genbank/). Branches with asterisks indicate statistical support from SH-test, aBayesian test, maximum-likelihood bootstrap and internodes certainty ≥90, 0.90,90 and 0.90 respectively. The tree was rooted by Oedogoniales. Clades with present OTUs were colored in red

All Chlorophyceaen OTUs were nested into the *Jenufa* clade (incertae sedis) and in the Volvocales. And those OTUs did not form monophyletic group but clustered with their relatives retrieved from database (Fig. 4). 22 and 6 OTUs were annotated as *Jenufa* clade and Volvocales respectively (2 in *Reinhardtia* clade and 4 in *Stephanosphaerinia* clade). There were 11 and 9 OTUs annotated as Dinophycean and Eustigmatophycean groups, respectively. Both Dinophycean and Eustigmatophyceaen OTUs formed monophyletic groups sister to Gymnodiniales sensu stricto and *Monodopsis* (Monodopsidaceae) respectively (Fig. 5).

### Composition of algal communities from the phyllosphere in the studied area

**Fig. 6:**
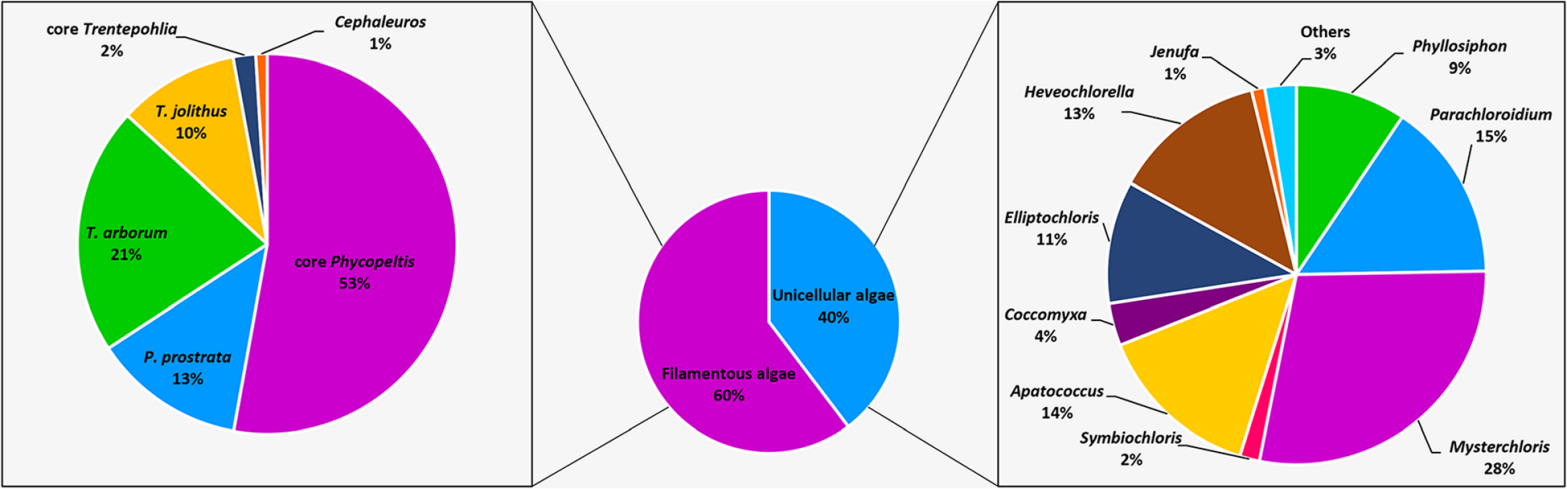
Taxonomic structure and diversity of the reads mapping to eukaryotic algae assigned at genus-level

Among the 2922 OTUs from all samples, filamentous forms (Trentepohliales) represented the highest percentage of taxa, about 60% with 1790. In the unicellular group (1132 OTUs), the *Mysteriochloris* group had the highest percentage (28%), followed by the *Parachloroidium* group (15%), *Apatococcus* group (14%), *Heveochlorella* group (13%), *Elliptochloris* group (11%), *Phyllosiphon* group (9%), *Coccomyxa* group (4%), *Symbiochloris* group (2%), and *Jenufa* group (1%) (Fig. 6).

**Fig. 7:**
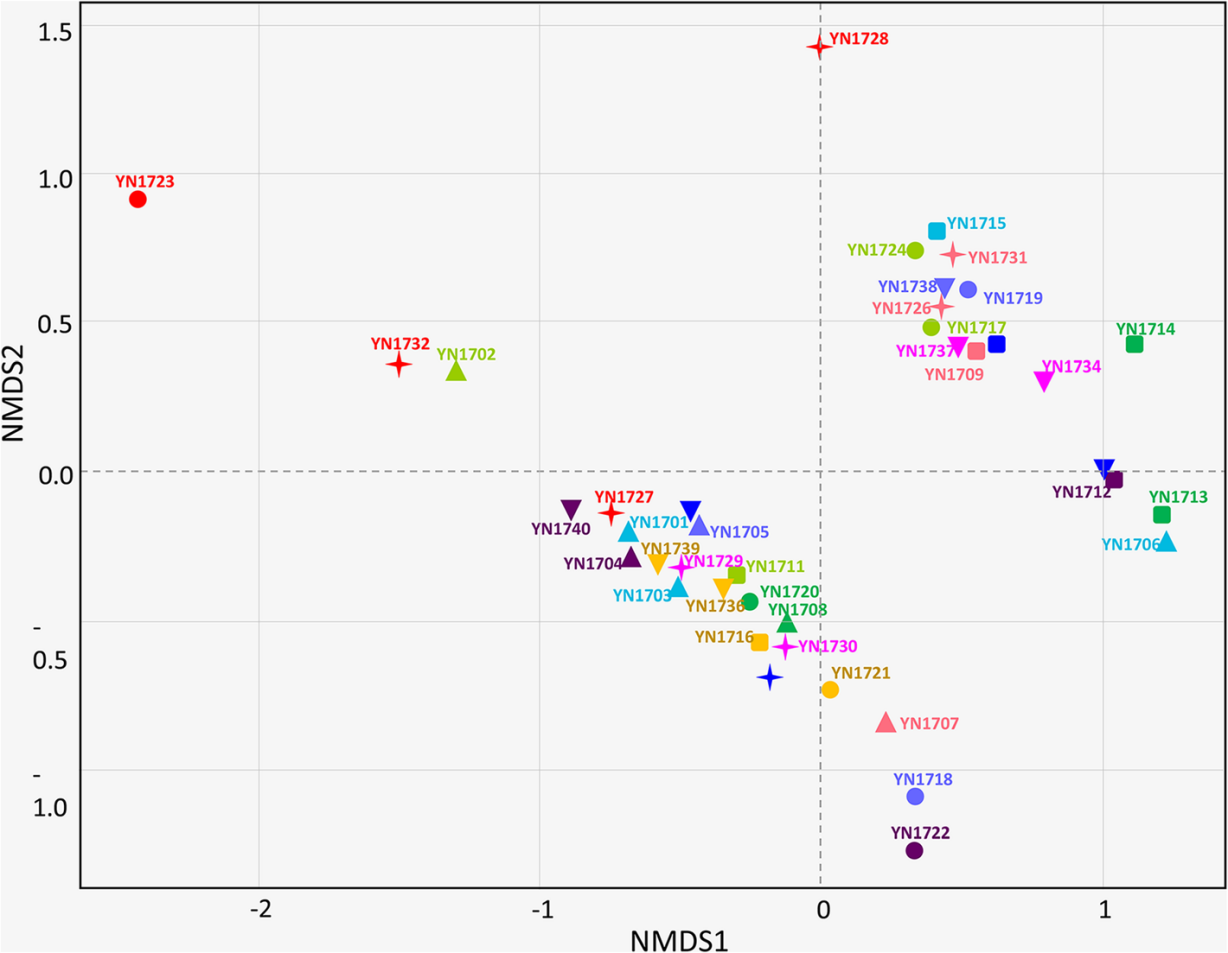
Grouping of the 40 phyllosphere algal communities according to species-level taxonomic compositional similarity (Bray-Crutis distances) using nonlinear multidimensional scaling. Each symbol or color represents one sampling location or host tree

The alpha diversity of all 40 samples were showed in Additional file 3: Figure S3, including richness (number of OTUs), Shannon diversity and Simpson diversity. The number of observed OTUs and algal abundance among 10 host trees were 566 to 934 and 7248 to 12,466, respectively (Additional file 4: Figure S4), and among 5 different sampling locations were 1085 to 1321 and 17,983 to 22,844 (Additional file 5: Figure S5). The ANOVA analysis and nonmetric multidimensional scaling (NMDS) analysis showed the alpha-diversity and beta-diversity of algal community structures at species level has no significant difference between different host trees and different sampling locations (Table 1 and Fig. 7).

**Table 1:** Two-way ANOVA analysis of effects of host species and sampling locations. (P < 0.05 was considered as statistically significant)

	Host species		Location		Species*Location	
	F	p	F	p	F	p
Shannon index	1.29	0.34	2.56	0.09	1.46	0.26
Simpson index	0.63	0.75	1.65	0.23	1.23	0.37
Richness	1.82	0.17	1.86	0.18	0.8	0.66
Abundance	1.69	0.2	1.5	0.26	0.79	0.67

**Fig. 8:**
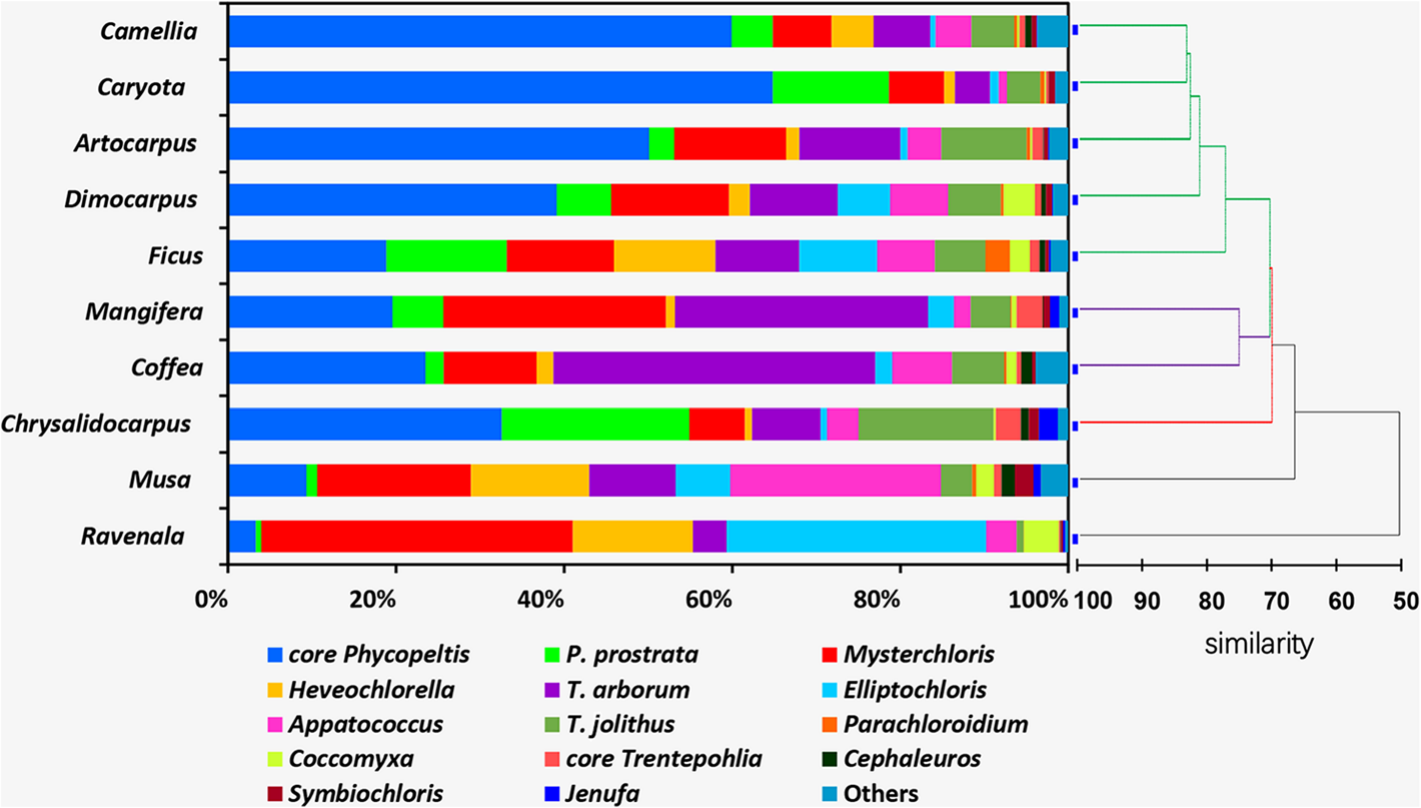
Comparison of eukaryotic algal reads distribution (assigned at the geneus-level) for 10 different host trees and the comparison of eukaryotic algal community structures from 10 host trees at the genus level based on hierarchical clustering. The dissimilarity matrix was computed using Bray-Crutis distance. The colored lines indicated Similarity Profile Analysis result with significance (*P* < 0.05)

Similarity Profile Analysis results showed that the ten communities at genus level formed five clades, three of which were significantly supported (*P* < 0.05, in the color lines, Fig. 8). Obviously, most host trees have a community dominated by algae of the core *Phycopeltis* clade, namely, *Camellia*, *Caryota*, *Artocarpus*, *Dimocarpus* (in green lines); *Mangifera* and *Coffea* have a community in which the *Trentepohlia arborum* clade and the core *Phycopeltis* clade are dominant. *Musa* and *Ravenala* differed from other trees in having a community dominated by coccoid algae (i.e., *Mysteriochloris* clade, *Heveochlorella* clade, *Apatococcus* clade and *Elliptochloris* clade); in these two plants Trentepohliales had a very low abundance.

**Fig. 9:**
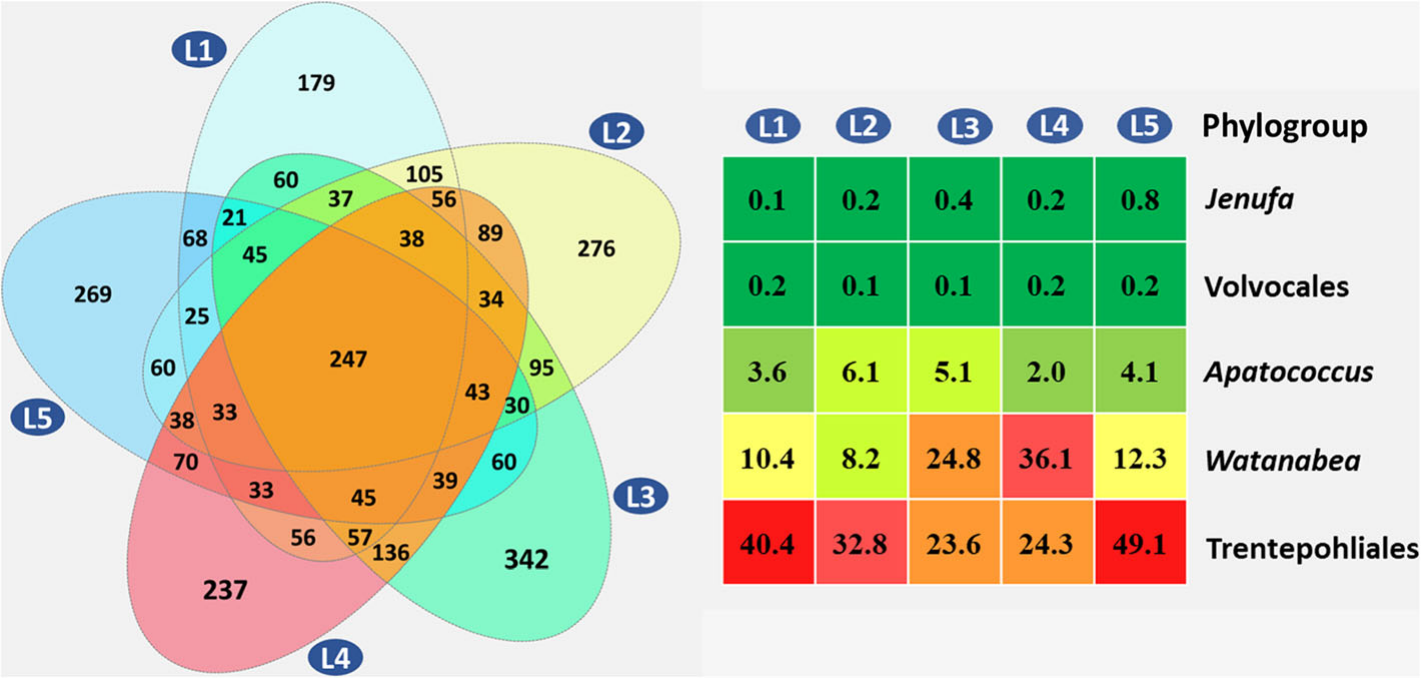
The extent of overlap of algal communities between 5 locations showed in Venn diagram (left). And the abundance of the core algal microbiome, colors from green to red represent their abundance (right)

In present study, we detected a core phyllosphere algal microbiome of common and abundant eukaryotic algae taxa present at all sites. The venn analysis showed that 56.45% (1689 OTUs) of the unique sequences were detected at two sites (Fig. 9), and only 8.26% (247 OTUs) were found at all 5 sampling sites (Fig. 9). These 247 OTUs were assigned to five clades, i.e., Trentepohliales clade, *Watanabea* clade, *Apatococcus* clade, *Jenufa* clade and Volvocales clade. It could be easily found that Trentepohlialean OTUs and *Watanabea* OTUs had the highest abundance followed by *Apatoccus* clade OTUs, whereas OTUs of the *Jenufa* clade and Volvocales were lowest (Fig. 9). Thus, we concluded that the core algal microbiome of the phyllosphere in the study area mainly consisted of algae belonging to the Trentepohliales, *Watanabea* clade and *Apatococcus* clade.

## Discussion

A common identity threshold that has been used to delimit OTUs is 97%; this threshold, however, has been mostly used for sequences of the 18S rDNA V4 or V9 regions with a length about 150–450 bp [35, 36]. The present study used sequences covering almost the whole length 18S rDNA gene, which in our opinion makes our results more discriminating than results based only on the V4 or V9 region. We agree with previous studies remarking that a 98% identity threshold should provide a better taxonomic resolution that allows to investigate the interspecific diversity when using full 18S rDNA sequences [37].

The present study examined 40 algal communities from ten different tropical host trees using nearly whole length of the 18S rDNA gene and provided an accurate characterization of the eukaryotic algal diversity of these communities. Our annotation results and phylogenetic results recovered five classes of eukaryotic algae in the phyllosphere, i.e., Trebouxiophyceae, Chlorophyceae, Ulvophyceae, Eustigmatophyceae and Dinophyceae. Many genera and species of Trebouxiophyceae, Trentepohliales and Eustigmatales are known predominantly from diverse aerial habitats, i.e., artificial substrates, tree trunks, leaves, wet soils and even desert soils [11, 14, 26, 38–41]. As one of the least known algal habitats world-wide, the identification of those group from phyllosphere is difficult due to their uncertainties in taxonomy and large cryptic diversity [2]. The possibly undescribed and unidentified green algae in tropical forests could be about 60% according to traditional morphological criteria [17]. According to our results, it is likely that this percentage may be far higher than 60%. For example, there are about 80 species currrently recorded for the Trentepohliales and there are about 26 species in the *Watanabea* clade as defined on morphological basis (belonging to the genera *Watanabea*, *Desertella*, *Heveochlorella*, *Heterochlorella*, *Phyllosiphon*, *Chloroidium*, *Parachloroidium*, *Polulichloris*, *Kalinella* and *Mysteriochloris*) (http://www.algaebase.org; searched on May, 2018). Our results unravel a much higher diversity of 18S rDNA than expected in phyllosphere community, especially in *Watanabea* clade and Trentepohliales.

The order Trentepohliales is a group of strictly subaerial algae that, besides free-living forms, include epiphytic or endophytic forms as well as lichen phycobionts. Recent phylogenetic studies of this group recovered at least seven lineages i.e., *Cephaleuros*, *Stomatochroon*, *Trentepohlia arborum* clade, *T. aurea* clade, core *Phycopeltis* clade, and several other *Trentepohlia* clades (such as *T. jolithus* clade, *T. umbrina* clade and *T. annulata* clade). Our knowledge about the phylogeny of Trentepohliales so far has been substantially limited by the fact that most sequence data come from cultured strains and limited environmental DNA sequences [42]. Many species of the order cannot be successfully cultured in the lab (especially of the genus *Phycopeltis*, which is mostly epiphytic on leaves). Our result once again indicates that *Phycopeltis* and *Trentepohlia* are polyphyletic. Additionally, our phylogenetic analysis demonstrates that the seven lineages so far discovered represent only a very small part of the trentepohlialean 18S rDNA diversity. Our analysis recovered several lineages previously not identified in 18S rDNA phylogenies; we consider that these lineages are likely to include undescribed species. Further studies of on foliicolous Trentepohliales based on methods such as single cell PCR may allow to get new insights and reassess the classification of this order.

Many of Trebouxiophycean algae are reported from terrestrial environments or as symbionts and endosymbionts with independent evolutions, i.e., *Lunachloris*, *Choricystis*, *Trebouxia*, *Symbiochloris* and *Xylochloris* [19, 43–45]. Molecular phylogenies of this class recovered four orders (Chlorellales, Trebouxiales, Prasiolales, Microthamnionales) and several not well-defined clades, i.e., *Neocystis*-*Coenocystis* clade, *Apatococcus* clade, *Coccobotrys* clade, *Choricystis*-*Botryococcus*-*Coccomyxa* clade, *Elliptochloris* clade, *Eremochloris* clade, *Xylochloris* clade and *Watanabea* clade [11, 22, 45, 46]. Nearly all these distinct lineages and orders include genera or species from terrestrial habitats. The Trebouxiophycean OTUs in the present study were distributed in 6 main clades (consisted of 43 well supported small clades). The many species of the genera *Stichococcus* and *Apatococcus* are frequently encountered in aerial habitats [47–49], and species of *Trebouxia*, *Elliptochloris* and *Symbiochloris* were described as symbionts of epiphytic lichens [44, 50, 51], so it is not unexpected that we obtained OTUs referable to these genera or clades. Although for some genera and species of Trebouxiophyceae the classification is still confused, the discovery in the present study of new clades in *Symbiochloris*, *Elliptochloris* and the Trebouxiales clades leads us to suspect that the molecular diversity in these taxa is far higher than appreciated so far.

The *Watanabea* clade as morphologically circumscribed includes genera mainly found in aerial habitats such as surface of tree bark or leaves, i.e., *Watanabea*, *Viridiella*, *Chloroidium*, *Parachloridium*, *Heveochlorella*, *Heterochlorella*, *Kalinella*, *Polulichloris* and *Mysteriochloris* [20, 52–57], most of which with limited 18S rDNA sequences published in database (such as genera *Mysteriochloris*, *Polulichloris* and *Kalinella*). According to our results, the *Symbiochloris* clade also falls into the traditional *Watanabea* clade, in contrast to previous studies [44]. The bulk of novel sequences in the present study have a maximum similarity from 89 to 95% with published sequences of traditional *Watanabea* clade, especially the genera *Mysteriochloris*, *Phyllosiphon* and *Heveochlorella*. Thus, the large genetic distance rendered the traditional order level *Watanabea* clade and its sub-branches such as *Mysterchloris*, *Phyllosiphon*, *Parachloroidium* and *Heveochlorella* clades should be re-evaluated in further study.

It was not surprising that some *Jenufa* clade and Volvocales sequences were discovered in our study, since species of *Jenufa* are recorded from tropical or subtropical forests [18, 58] and *Protosiphon* species (fall into *Stephanosphaerinia* clade) are also reported from aerial habitats such as soils [40, 59]. There are only 4 species in *Jenufa*, and only 2 OTUs have a 100% similarity with published sequences. Most OTUs are similar with those published sequences at 95–99% level, which indicates that *Jenufa* clade need further deep diversity investigation.

It has been reported that heterokont algae such as Xanthophyceae and Eustigmatophyceae occur in aerial habitats, i.e. surface of rocks or corticolous communities of forest, free-living or lichenized [43]. Eustigmatophyceae have been rarely recorded, and usually thought to comprise a few genera and species belonging to two orders, Eustigmatales and Goniolchloridales [60]. The present environmental sequences formed a new clade sister to *Monodopsis*, demonstrating that the eustigmatophyceaen algae may have a large distribution and require further diversity investigation. The record of dinoflagellates sequences from tropical forest leaves was an unexpected result. Surprisingly, this is the first record of dinophytes from tropical forest leaves. These foliicolous dinoflagellate may represent a new group, since the 11 dinophyte OTUs formed a clade closely related to Gymnodiniales sensu stricto in the present study.

We did not detect any OTUs referable to another “flagship” aerial group, the Klebsormidiales, algae which are commonly found on most aerial surfaces and thrive on artificial substrates such as old walls [29, 47]. Species of Klebsormidiales have been rarely reported from tropical forest leaves. Maybe species of this group has no advantages in tropical phyllosphere algal community.

To our knowledge, this is the first study providing a comprehensive comparison of foliicolous algal communities based on a large-scale molecular dataset. Although the sampling sites were circumscribed to one forest garden, the eukaryotic algal diversity revealed through the 18S rDNA gene in this environment is clearly very high. Usually coccoid algae do not occur on leaf surfaces as solitary cells but as aggregates, a situation very similar to bacteria [1]. The influence of the host trees on the fungal and bacterial communities is well known, and studies on these communities showed that the plant species is an important determinant [7, 61]. Many studies proved that the abundance of bacteria and fungi was affected by plant species [61–63]. In contrast, the composition of the algal communities in terms of species/taxa (with OTU identity cutoff 98%) is not significantly different in relation to host trees and locations according to our result.

Obviously, most host trees have a community dominated by Trentepohliales, in contrast, *Musa* and *Ravenala* have a coccoid green algae-dominant (especially *Mysteriochloris* and *Elliptochloris* and *Heveochlorella*) community. Thus, there is no significant difference among the community structure of *Caryota*, *Chrysalidocarpus*, *Camellia*, *Artocarpus*, *Coffea*, *Mangifera* and *Dimocarpus* either at species level or order level. However, community profiling at family or order level (same as clades defined in present study) showed striking differences between *Musa*, *Ravenala* and the other trees (Additional file 6: Figure S6). Since the eight *Musa* and the eight *Ravenala* were sampled from different sites, this does not seem to be a difference related to different sites. However, since we did not get information about host functional traits (such as leaf growth rate, concentration of metal ion, phosphorus and nitrogen) and the physiological requirements of those algae, it is hard to explain the correlation between Trebouxiophycean algae and *Musa* and *Ravenala*. Plants such as *Musa* and *Ravenala* have a cuticle that is thinner than other plants such as *Caryota*, *Coffea*, *Dimocarpus* and so on. Such leaves surfaces of *Musa* and *Ravenala* are not suitable for the survival of *Phycopeltis* because *Phycopeltis* is mainly found on very smooth surfaces such as leaf with thick cuticle (leathery leaf) and other abiotic substratum (plastic tags, i.e.), which leads to a community with dominant coccoid algae.

Previous studies reported that phylogenetic distance does not predict competition in the green algal communities, either in natural or experimental ones, which challenged the competition-related hypothesis [64, 65]. Although, such inference was concluded from phytoplankton communities, it seems also coincident with our observation of phyllosphere communities. Our result showed that interspecies diversity on same leaves was very rich, which, on the other hand, may predict that the strength of competition is not strong. Phylogenetic analyses of certain algae and their nearest relatives allow exploration of the number and rapidity of transitions to the habitat of interest and can provide insights into the physiological traits important in these transitions [39]. In present study, it has reported that algae in the *Watanabea* clade were reported to contain several mycosporine-like amino acids (MAAs) for surviving from excessive ultra-violet radiation [66]. Future studies on the physiology of those algae may provide more clues to understand their adaptations to the phyllosphere.

## Conclusions

SMRT sequencing is very useful for accurately profiling the phyllosphere algal communities. Our results showed that the diversity of eukaryotic algae is very high in tropical phyllosphere. There are still many undescribed species in such habitat, especially in Chlorophytes group. Trentepohliales, *Watanabea* clade and *Apatococcus* clade are the dominant algae in foliicolous communities from tropical forest. The community structure at the species level does not have a significant relationship either with their host plants or locations. *Musa* and *Ravenala*, with the leaf texture and growth rate different from other plants, differed from other host plants significantly at the genus level, since they were dominated by Trebouxiophycean epiphytes. The present study provided a large amount of novel 18S rDNA sequences that will be useful to unravel the cryptic diversity of phyllosphere eukaryotic algae and for comparisons with similar future studies on this type of communities.

## Methods

### Sampling sites and collection

**Fig. 10:**
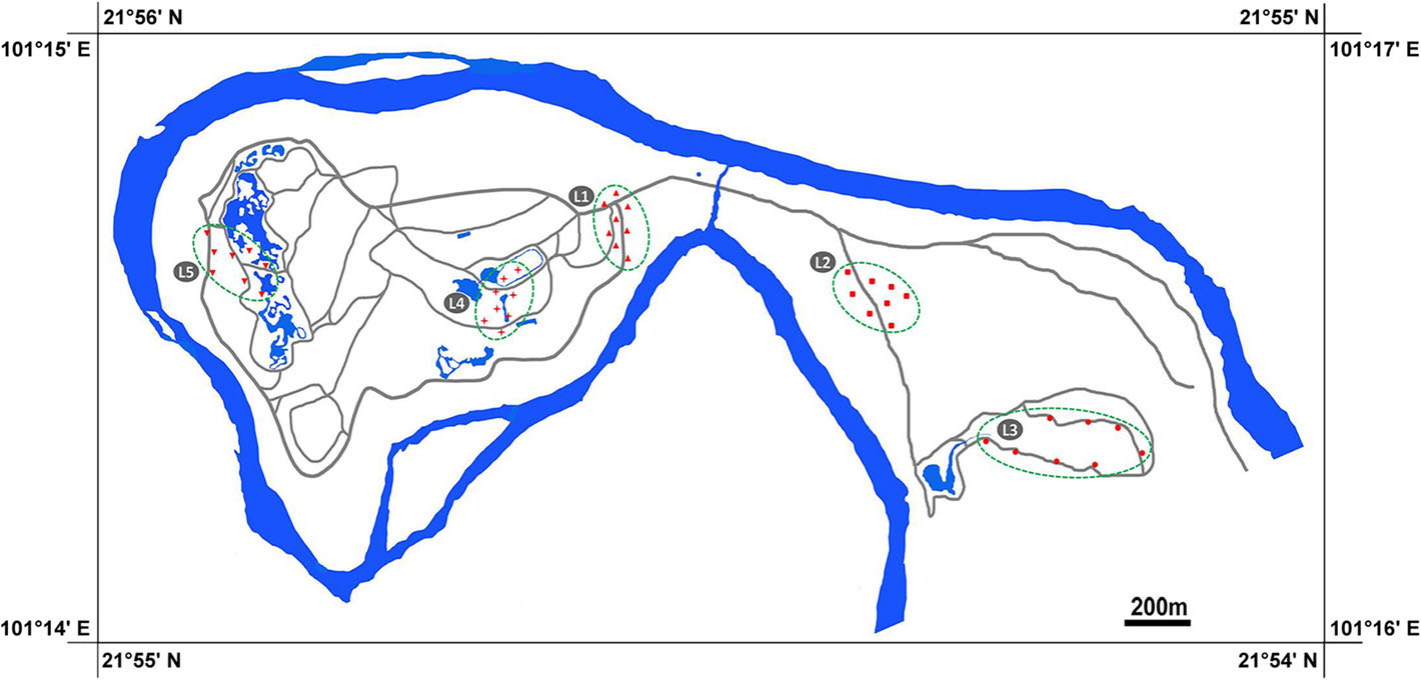
Map of 5 sampling locations and 40 sampling sites from Xishuangbanna Tropical Botanical Garden (The map was drawn by the authors based on the information from National administration of surveying, mapping and geoinformation, http://www.sbsm.gov.cn/). Detailed sampling sites can be viewed at Additional file 7: Table S1

We collected eukaryotic algae from the leaf surfaces of land plants randomly in Xishuangbanna Tropical Botanical Garden in August 2017. The samples were collected from 5 sites (Fig. 10 and Additional file 7: Table S1); we collected 8 samples from each site. Each sample was collected at a distance of at least 10 m from all other samples. We selected ten different host trees (*Mangifera*, *Artocarpus*, *Dimocarpus*, *Ficus*, *Coffea*, *Camellia*, *Chrysalidocarpus*, *Caryota*, *Musa* and *Ravenala*). Each sample was collected 2 m above ground (thus we could not observe the positive surface to make sure all leaves were collected randomly) by clipping 2–6 leaves from an individual plant into Kraft paper bags from different site and different host tree. A total of 40 samples were collected in present study and brought to laboratory. The observable foliicolous masses were scraped slightly under a stereoscope using a small scalpel and a brush. Leaves devoid of evident algal coverage were cut into small fragments and shaken in small bags with diluted PBS (Phosphate Buffered Saline, pH = 7.4), then the microbial mass was pelted by centrifuging at 6000×g for 10 min. For each sample, a part of algal mass was dried by a vacuum freeze dryer and stored at − 80 °C. The remainders of the samples were dried using specimen clip and deposited in the Freshwater Algal Herbarium, Institute of Hydrobiology, Chinese Academy of Sciences, Wuhan.

### DNA extraction, amplification and sequencing

The total DNA of each environmental mass (about 0.05 g) was extracted using the Omega higher plant DNA kit following its manual. We used two couples of universal SSU rDNA primers, NS1F and NS8R, NS1F and 1650R [67] to amplify the SSU rDNA gene. For both couples of primers, the thermal-cycling profiles were as follows: 94 °C for 5 min, then 33 cycles of 94 °C for 30s, 54 °C for 45 s and 72 °C for 60s, followed by a final extension at 72 °C for 6 min. The products of each sample were mixed and purified with the QiaEX Gel Extraction Kit (Qiagen, Valencia, CA, USA) and sent to Shanghai Personal Biotechnology Co., Ltd. for SMRT sequencing by Pacbio Squeal platform. The products of each sample were assigned to unique forward and reverse barcode respectively, and then just one library was constructed for sequencing. We used circular consensus sequencing approach (CCS). With consensus sequencing at least three passes (≥3 CCS), the average error rate was guaranteed to decrease theoretically less than 1.0‰. All sequences have been deposited in the SRA of the NCBI database under the accession no. SRP14140.

### Bioinformatic analysis

Raw reads without adapter were assigned to different samples based on the barcodes (Additional file 8: Table S2). Any joined sequences with ambiguous bases (including sequences with more than 5 mismatches base-pairs at 5′ end and with more than 8 same sequential base-pairs and Chimera sequences) and lengths < 1500 bp were discarded by QIIME (Quantitive Insigts Into Micriobial Ecology, v1.8.0) [68]. High-quality sequences were further processed using mothur (v. 1.34) following the recommendations by Schloss et al. [69]. Sequences were clustered into operational taxonomic units (OTUs) at a 98% identity threshold. The representative sequences of each OTU were annotated by BLASTn, and only eukaryotic algal OTUs were processed consequently. All the representative sequences were assigned to five algal classes, namely Trebouxiophyceae, Ulvophyceae, Chlorophyceae, Dinophyceae and Eustigmatophyceae. OTUs with abundance lower than 0.001% were removed from the community data matrix but were retained for phylogenetic analysis.

### Taxonomic analysis of all OTUs

Five matrices (Trebouxiophyceae, Ulvophyceae, Chlorophyceae, Dinophyceae and Eustigmatophyceae) were constructed by OTUs and tentative closely related sequences downloaded from GenBank (https://www.ncbi.nlm.nih.gov/genbank/). The matrices were aligned by mafft 7.3 [70]. The best model for each matrix was selected using Modelfinder according to AIC criteria [71]. In present study, we performed Maximum Likelihood analysis by IQ-TREE [72] and used SH test and Internodes Certainty to evaluate the topology of the Maximum Likelihood tree. The UFboot2 method was used to perform bootstrap analyses under best-fit model [73]. For all five UFboot2 analysis, Bootstrap correlation coefficient of split occurrence frequencies was set to 0.99. Then the Internodes Certainty was tested using bootstrap trees obtained from IQtree in RaxML [74].

According to results of the phylogenetic inference, we divided nearly all OTUs into 11 order-level phylogroups (named as *Apatococcus* group, *Watanabea* group, *CBCE* group, Prasiolales group, Trebouxiales group, Microthamnionales group, Volvocales group, *Jenufa* group, Trentepohliales group, Eustigmatales group and Gymnodiniales group) for subsequent ecological analyses. OTUs were subdivided into two morphotypes, filamentous algae and unicellular algae.

### Statistical analysis

Alpha diversity including the Shannon-Wiener index and Simpson’s diversity index were calculated for each sample using the QIIME software. The venn analysis was performed in Venndiagram package [75] in R version 3.2 [76]. A two-way Analysis of Variance (ANOVA) was performed to test the effects of host trees and sampling sites on alpha diversity (number of OTUs, Shannon-Wiener index and Simpson’s index) and the relative abundances of different algal taxa. ANOVA was performed using SPSS 18.0 package (SPSS, USA), with a *p*-value < 0.05 selected for significance. The original data were normalized using standardization prior to statistical analysis and ANOSIM (Analysis of Similarities), SIMPROF (Similarity Profile Analysis) and NMDS (Non-parametric Multi-Dimensional Scaling) were performed using Primer 6.0 [77]. The resemblance of data matrix was measured by Bray-Curtis dissimilarity.

## Additional files

Additional file 1: **Figure S1.** Lengths distribution of quality sequences. The frequency of the sequences length (in base-pairs) is plotted for the 152,324 sequences. (TIF 944 kb)

Additional file 2: **Figure S2.** Rarefaction analysis showing sampling intensity of 40 samples. Random sub-samplings were conducted for sequencing depth from 0 to 700 sequences. (TIF 1261 kb)

Additional file 3: **Figure S3.** The alpha diversity of 40 samples, including number of OTUs, Shannon diversity and Simpson diversity. (TIF 1927 kb)

Additional file 4: **Figure S4.** The alpha diversity of 10 host trees, including Richness (number of OTUs), Abundance (number of sequences), Shannon diversity and Simpson diversity. (TIF 705 kb)

Additional file 5: **Figure S5.** The alpha diversity of 5 sampling locations, including Richness (number of OTUs), Abundance (number of sequences), Shannon diversity and Simpson diversity. (TIF 541 kb)

Additional file 6: **Figure S6.** Nonlinear multidimensional scaling analysis of 40 phyllosphere algal community structures according to order-level taxonomic compositional similarity (Bray-Crutis distances). (TIF 850 kb)

Additional file 7: **Table S1.** Sampling sites of the 40 leaves specimens from Xishuangbanna Tropical Botanical Garden and quantity of obtained sequences from each sample. (DOCX 18 kb)

Additional file 8: **Table S2.** The 40 barcodes used to identify samples within the pooled sequencing run. (DOCX 15 kb)

## Abbreviations

ANOSIM: Analysis of similarities
ANOVA: Analysis of variance
CCS: Circular consensus sequencing
MAAs: Mycosporine-like amino acids
NMDS: Non-parametric multi-dimensional scaling
OTUs: Operational taxonomic units
SIMPROF: Similarity profile analysis
SMRT: Single molecule real-time sequencing
