# Supplementary figures and images for: Molecular characterization of eukaryotic algal communities in the tropical phyllosphere based on real-time sequencing of the 18S rDNA gene
Source: BMC Plant Biol. 2018 Dec 18;18:365. doi: 10.1186/s12870-018-1588-7 (PMC6299628; doi:10.1186/s12870-018-1588-7)

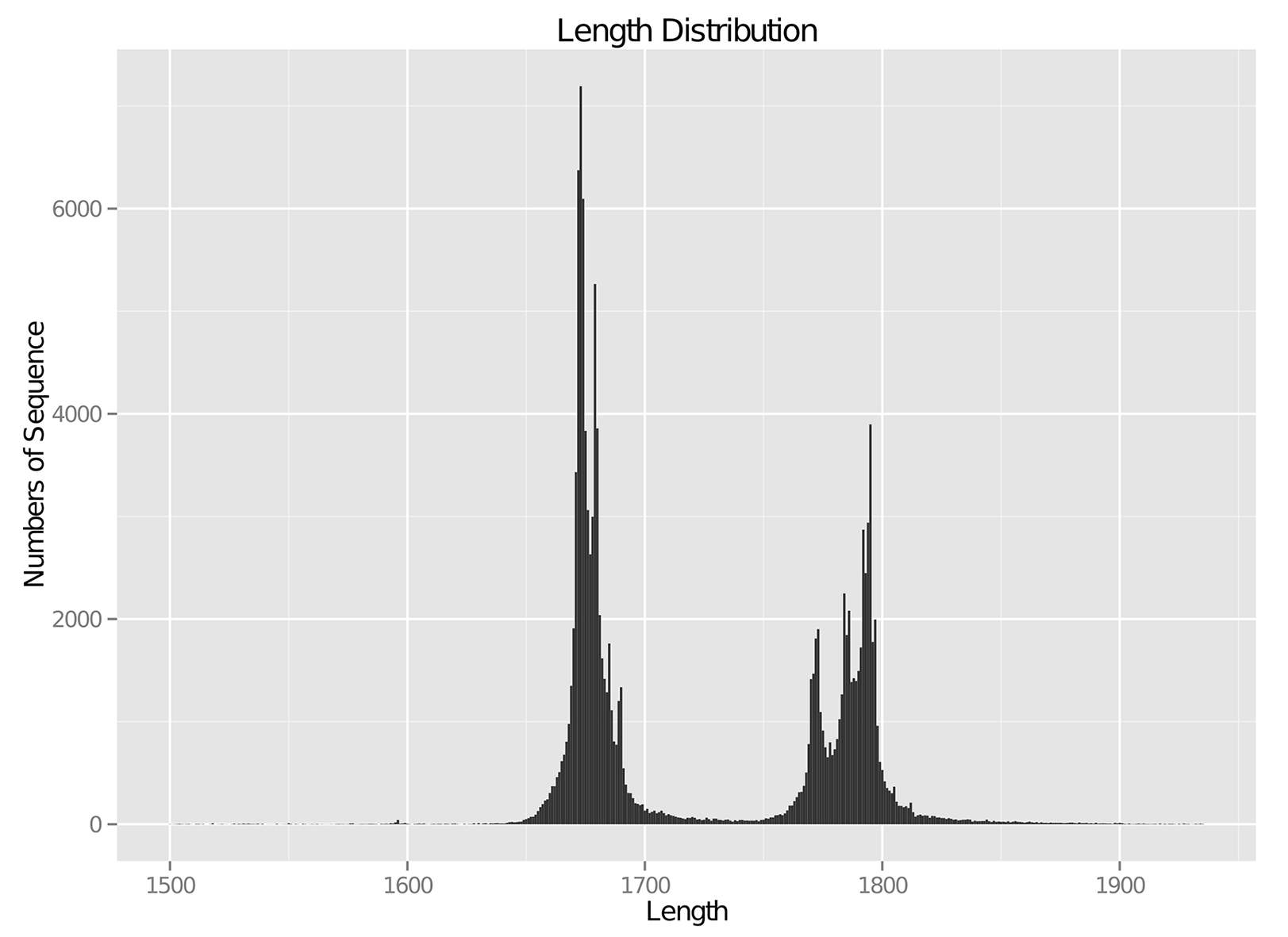

Supplement: Supplementary file 1 — Figure S1. Lengths distribution of quality sequences. The frequency of the sequences length (in base-pairs) is plotted for the 152,324 sequences. (TIF 944 kb) [file 12870_2018_1588_MOESM1_ESM.tif]

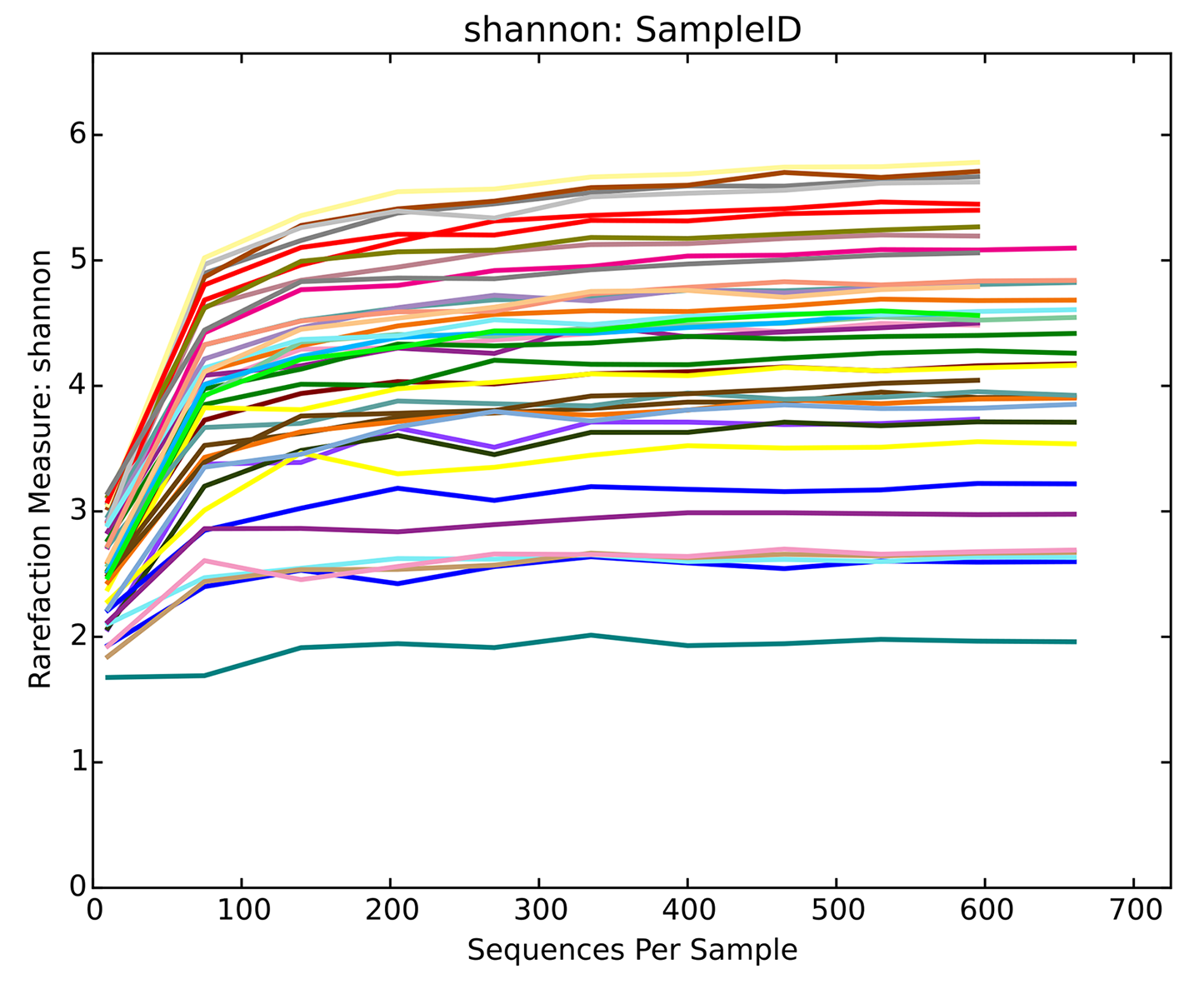

Supplement: Supplementary file 2 — Figure S2. Rarefaction analysis showing sampling intensity of 40 samples. Random sub-samplings were conducted for sequencing depth from 0 to 700 sequences. (TIF 1261 kb) [file 12870_2018_1588_MOESM2_ESM.tif]

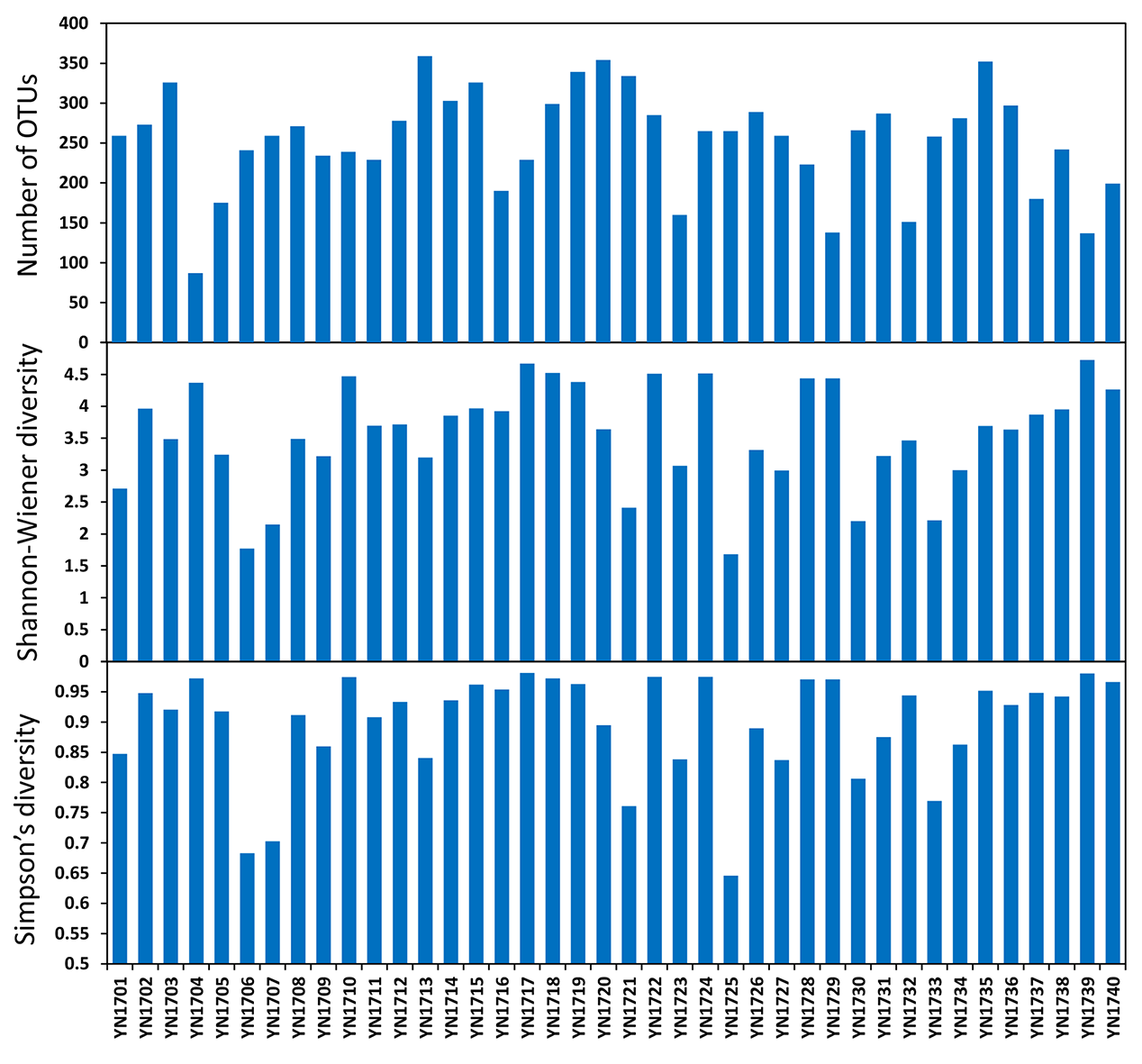

Supplement: Supplementary file 3 — Figure S3. The alpha diversity of 40 samples, including number of OTUs, Shannon diversity and Simpson diversity. (TIF 1927 kb) [file 12870_2018_1588_MOESM3_ESM.tif]

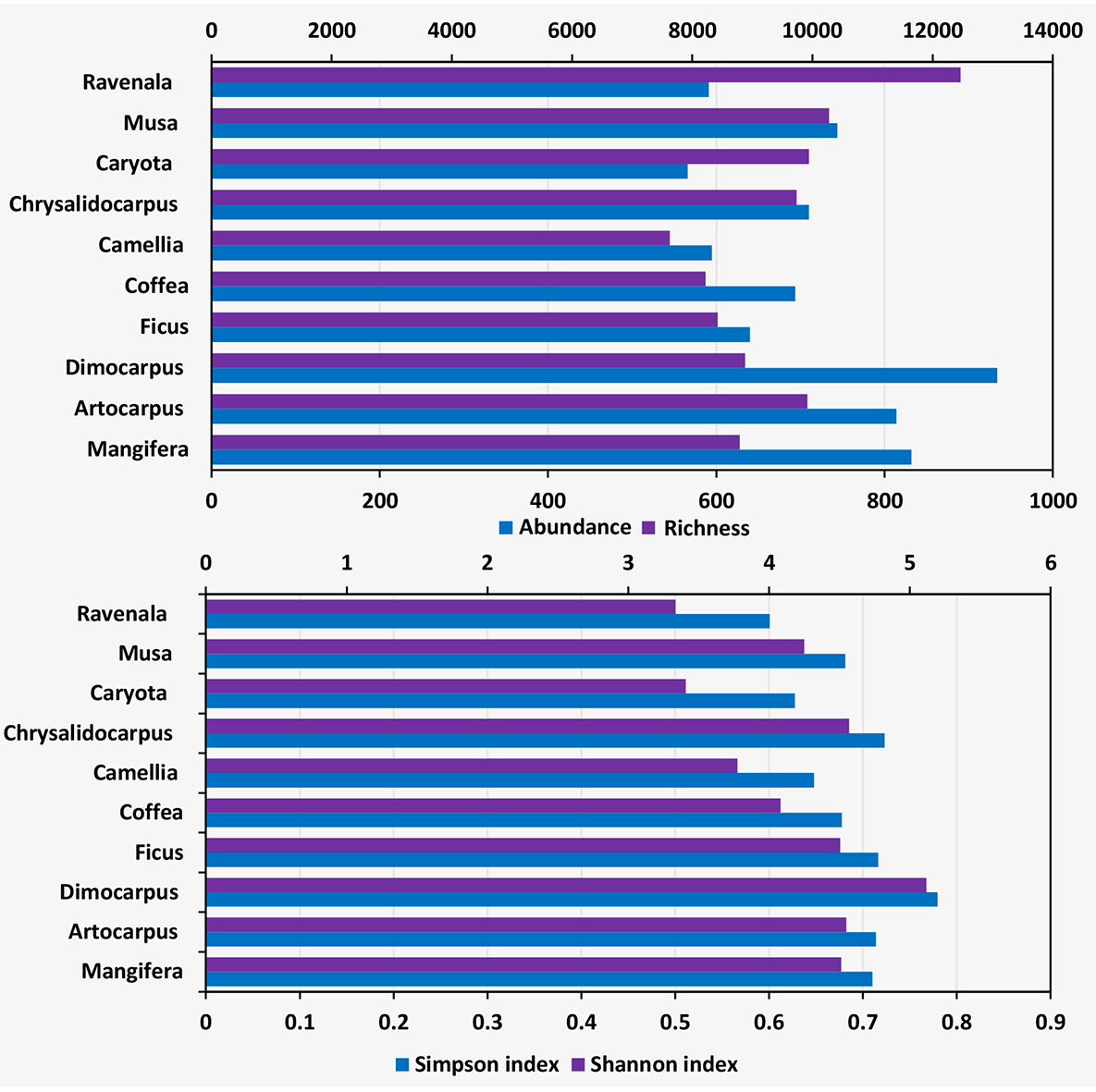

Supplement: Supplementary file 4 — Figure S4. The alpha diversity of 10 host trees, including Richness (number of OTUs), Abundance (number of sequences), Shannon diversity and Simpson diversity. (TIF 705 kb) [file 12870_2018_1588_MOESM4_ESM.tif]

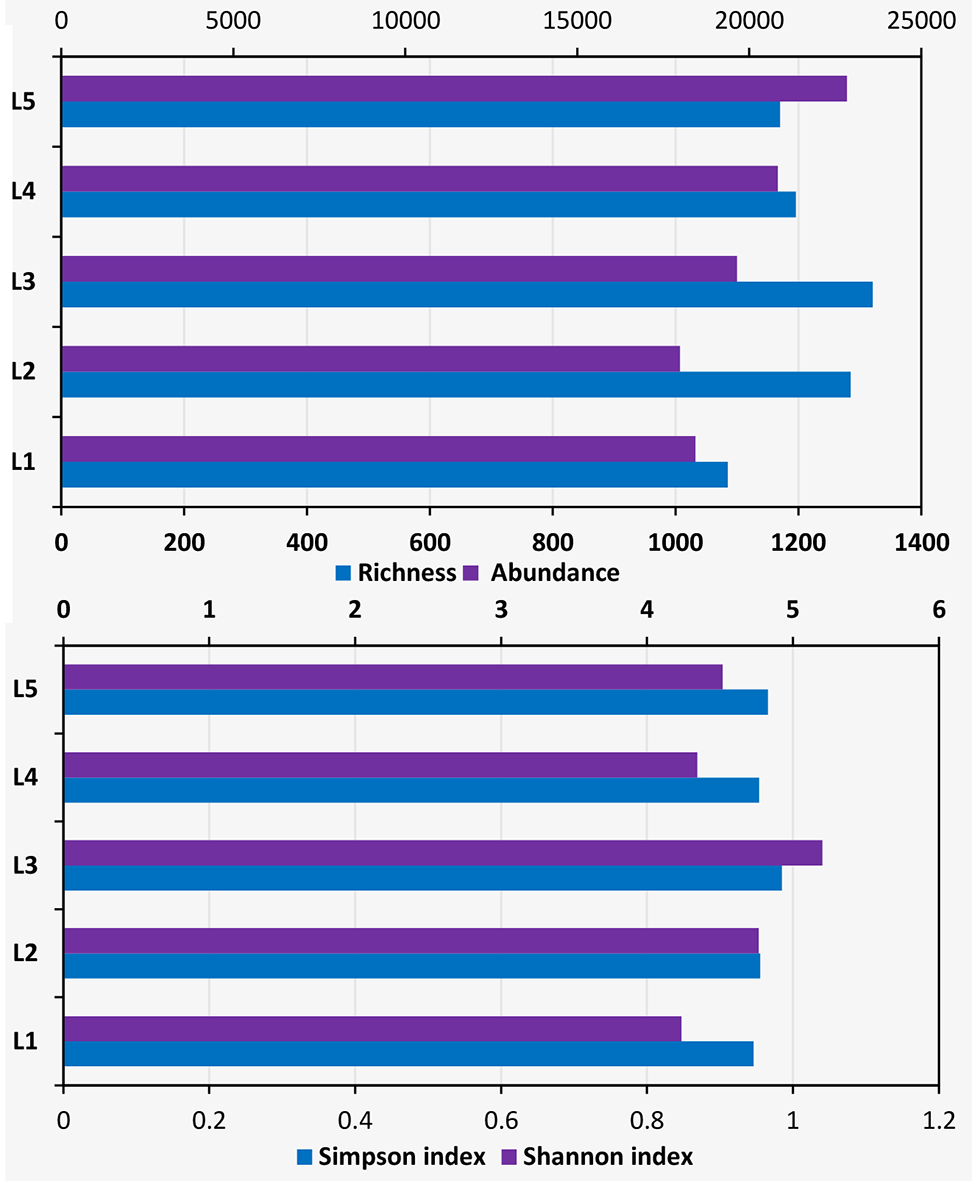

Supplement: Supplementary file 5 — Figure S5. The alpha diversity of 5 sampling locations, including Richness (number of OTUs), Abundance (number of sequences), Shannon diversity and Simpson diversity. (TIF 541 kb) [file 12870_2018_1588_MOESM5_ESM.tif]

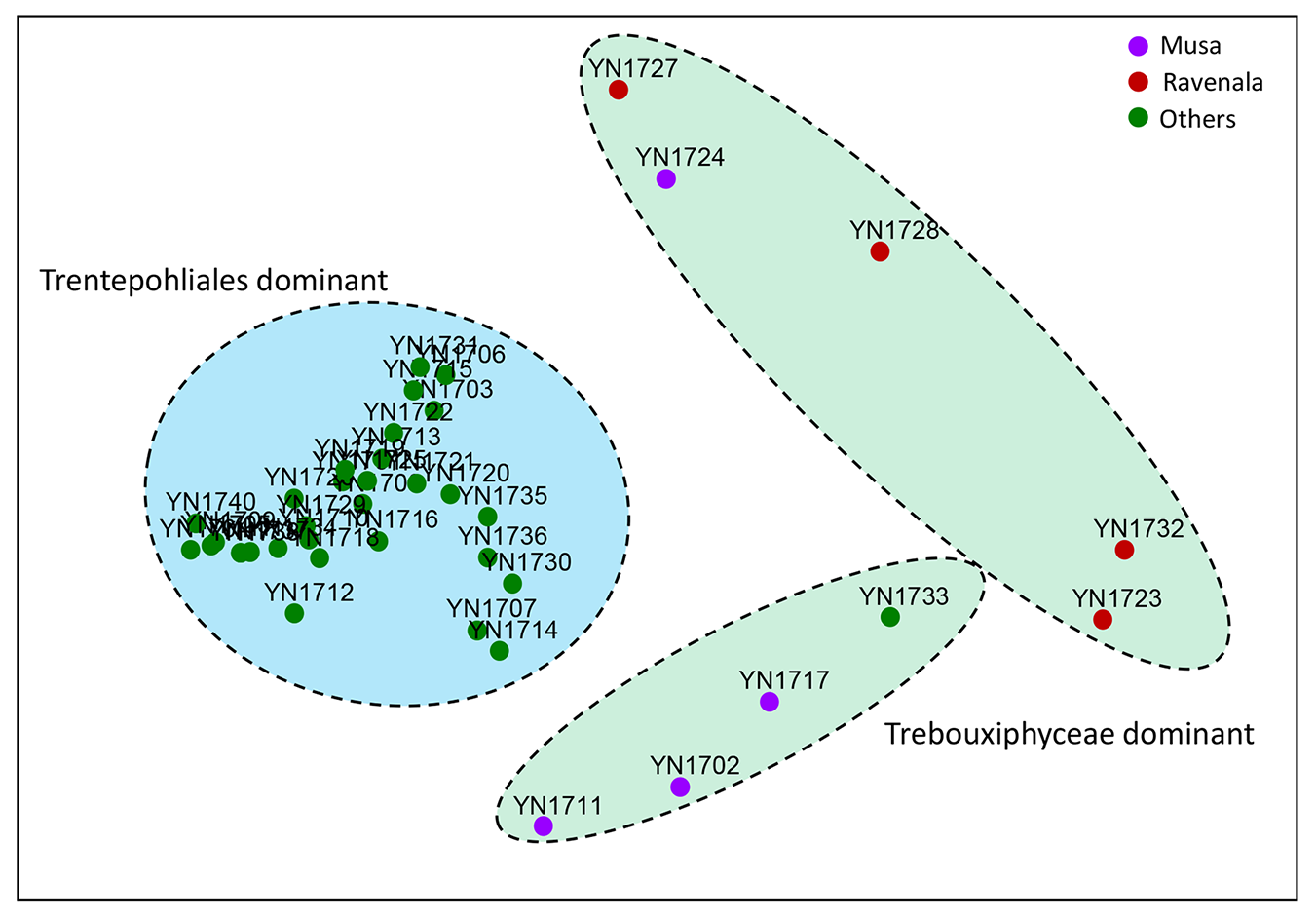

Supplement: Supplementary file 6 — Figure S6. Nonlinear multidimensional scaling analysis of 40 phyllosphere algal community structures according to order-level taxonomic compositional similarity (Bray-Crutis distances). (TIF 850 kb) [file 12870_2018_1588_MOESM6_ESM.tif]
