# Supplementary material for: Molecular characterization of eukaryotic algal communities in the tropical phyllosphere based on real-time sequencing of the 18S rDNA gene
Source: BMC Plant Biol. 2018 Dec 18;18:365. doi: 10.1186/s12870-018-1588-7 (PMC6299628; doi:10.1186/s12870-018-1588-7)
Supplement: Supplementary file 7 — Table S1. Sampling sites of the 40 leaves specimens from Xishuangbanna Tropical Botanical Garden and quantity of obtained sequences from each sample. (DOCX 18 kb) [file 12870_2018_1588_MOESM7_ESM.docx]

**Table S1** Sampling sites of the 40 leaves specimens from Xishuangbanna Tropical Botanical Garden.

| Sample | Longitude | Latitude | Locations | Host trees | Total sequences | Eukaryotic algae sequences |
| --- | --- | --- | --- | --- | --- | --- |
| YN1701 | 101°15'25"E | 21°55'43"N | L1 | *Mangifera* | 3538 | 2200 |
| YN1702 | 101°15'38"E | 21°55'42"N | L1 | *Musa* | 3368 | 2682 |
| YN1703 | 101°15'37"E | 21°55'42"N | L1 | *Mangifera* | 3637 | 2440 |
| YN1704 | 101°15'35"E | 21°55'40"N | L1 | *Chrysalidocarpus* | 3257 | 1711 |
| YN1705 | 101°15'31"E | 21°55'39"N | L1 | *Caryota* | 4832 | 3964 |
| YN1706 | 101°15'31"E | 21°55'40"N | L1 | *Mangifera* | 3231 | 2406 |
| YN1707 | 101°15'37"E | 21°55'39"N | L1 | *Artocarpus* | 3211 | 2316 |
| YN1708 | 101°15'37"E | 21°55'37"N | L1 | *Dimocarpus* | 4210 | 1913 |
| YN1709 | 101°16'01"E | 21°55'31"N | L4 | *Artocarpus* | 3772 | 3097 |
| YN1710 | 101°16'01"E | 21°55'29"N | L4 | *Ficus* | 4063 | 2316 |
| YN1711 | 101°16'02"E | 21°55'29"N | L4 | *Musa* | 2871 | 2775 |
| YN1712 | 101°16'03"E | 21°55'30"N | L4 | *Chrysalidocarpus* | 4181 | 2935 |
| YN1713 | 101°16'06"E | 21°55'28"N | L4 | *Dimocarpus* | 4525 | 2628 |
| YN1714 | 101°16'04"E | 21°55'29"N | L4 | *Dimocarpus* | 3426 | 2309 |
| YN1715 | 101°16'02"E | 21°55'29"N | L4 | *Mangifera* | 3102 | 1932 |
| YN1716 | 101°16'05"E | 21°55'25"N | L4 | *Coffea* | 2653 | 1787 |
| YN1717 | 101°16'09"E | 21°55'10"N | L3 | *Musa* | 5287 | 5128 |
| YN1718 | 101°16'10"E | 21°55'11"N | L3 | *Caryota* | 3828 | 2022 |
| YN1719 | 101°16'11"E | 21°55'12"N | L3 | *Caryota* | 4160 | 2516 |
| YN1720 | 101°16'10"E | 21°55'13"N | L3 | *Dimocarpus* | 4648 | 2625 |
| YN1721 | 101°16'10"E | 21°55'14"N | L3 | *Coffea* | 3619 | 1998 |
| YN1722 | 101°16'11"E | 21°55'13"N | L3 | *Chrysalidocarpu* | 3566 | 2202 |
| YN1723 | 101°16'12"E | 21°55'13"N | L3 | *Ravenala* | 3169 | 2370 |
| YN1724 | 101°16'12"E | 21°55'11"N | L3 | *Ravenala* | 3286 | 2154 |
| YN1725 | 101°15'24"E | 21°55'40"N | L2 | *Ficus* | 4207 | 2942 |
| YN1726 | 101°15'23"E | 21°55'39"N | L2 | *Artocarpus* | 3823 | 2869 |
| YN1727 | 101°15'21"E | 21°55'38"N | L2 | *Ravenala* | 5246 | 3821 |
| YN1728 | 101°15'24"E | 21°55'38"N | L2 | *Ravenala* | 3778 | 3182 |
| YN1729 | 101°15'21"E | 21°55'36"N | L2 | *Camellia* | 1098 | 556 |
| YN1730 | 101°15'22"E | 21°55'35"N | L2 | *Camellia* | 3088 | 2488 |
| YN1731 | 101°15'20"E | 21°55'35"N | L2 | *Artocarpus* | 3782 | 2013 |
| YN1732 | 101°15'21"E | 21°55'34"N | L2 | *Ravenala* | 5125 | 3728 |
| YN1733 | 101°14'47"E | 21°55'45"N | L5 | *Ficus* | 4789 | 4290 |
| YN1734 | 101°14'49"E | 21°55'48"N | L5 | *Camellia* | 3853 | 2583 |
| YN1735 | 101°14'49"E | 21°55'50"N | L5 | *Ficus* | 3734 | 2560 |
| YN1736 | 101°14'52"E | 21°55'48"N | L5 | *Coffea* | 4144 | 2204 |
| YN1737 | 101°14'55"E | 21°55'48"N | L5 | *Camellia* | 3642 | 2307 |
| YN1738 | 101°14'56"E | 21°55'47"N | L5 | *Caryota* | 4789 | 4107 |
| YN1739 | 101°14'53"E | 21°55'45"N | L5 | *Coffea* | 3488 | 2761 |
| YN1740 | 101°14'55"E | 21°55'44"N | L5 | *Chrysalidocarpus* | 4298 | 3281 |
