# Supplementary material for: Molecular characterization of eukaryotic algal communities in the tropical phyllosphere based on real-time sequencing of the 18S rDNA gene
Source: BMC Plant Biol. 2018 Dec 18;18:365. doi: 10.1186/s12870-018-1588-7 (PMC6299628; doi:10.1186/s12870-018-1588-7)
Supplement: Supplementary file 8 — Table S2. The 40 barcodes used to identify samples within the pooled sequencing run. (DOCX 15 kb) [file 12870_2018_1588_MOESM8_ESM.docx]

**Table S2** The barcodes used to identify samples within the pooled sequencing run.

| Samples | Barcodes | Samples | Barcodes |
| --- | --- | --- | --- |
| YN1701 | CGTAGCGTGCTATCAC | YN1721 | ACTGATCTGTCGCGCT |
| YN1702 | CACTAGCTCTGACTAC | YN1722 | ATGCTGATGACTGCGA |
| YN1703 | AGAGCATCTCTGTACT | YN1723 | GACTGAGATCATGATC |
| YN1704 | TGTGAGTCAGTACGCG | YN1724 | CATACGCTGTGTAGCA |
| YN1705 | CGAGAGTCAGCGCATA | YN1725 | AGCACTCGCGTCAGTG |
| YN1706 | TATCTATCGTATACGC | YN1726 | CGATCATCTATAGACA |
| YN1707 | GCTCGATCACATGACG | YN1727 | CAGTACTGCACGATCG |
| YN1708 | ACTCATATCTAGAGTG | YN1728 | ATACAGCACAGATGTG |
| YN1709 | TCACGATGAGCACGTA | YN1729 | TGCGTGAGCTGTACAT |
| YN1710 | CTGCTAGAGTCTACAG | YN1730 | GTCGTACACGTGCGAC |
| YN1711 | TACAGATAGTGTAGCG | YN1731 | TGTCGCAGCTACTAGT |
| YN1712 | TCATGAGTCGACACTA | YN1732 | ACGACATGATACTGCT |
| YN1713 | AGTCGCATGACTGTGT | YN1733 | ACAGTCGATATCTCTC |
| YN1714 | GAGCTGCGCACTCGAT | YN1734 | GTGCTGAGCATCAGAC |
| YN1715 | CACGTCACTAGAGCGA | YN1735 | GCGATGTCGCTATGTG |
| YN1716 | TCGTAGAGCTCGAGAC | YN1736 | ATCACACTGCATCTGA |
| YN1717 | ACGTACGCTCGTCATA | YN1737 | AGAGACACGATACTCA |
| YN1718 | TCATGCACGTCTCGCT | YN1738 | GCGCTCTGTGTGCAGC |
| YN1719 | CGACGTATCTGACAGT | YN1739 | TACAGTGTCTGCTGCG |
| YN1720 | CACTGATCGATATGCA | YN1740 | CGCATCGACTACGCTA |
